# The relationship between treatment-induced hypertension and efficacy of anlotinib in recurrent or metastatic esophageal squamous cell carcinoma

**DOI:** 10.20892/j.issn.2095-3941.2020.0187

**Published:** 2021-06-15

**Authors:** Yan Song, Juxiang Xiao, Wentao Fang, Ping Lu, Qingxia Fan, Yongqian Shu, Jifeng Feng, Shu Zhang, Yi Ba, Yang Zhao, Ying Liu, Chunmei Bai, Yuxian Bai, Yong Tang, Jie He, Jing Huang

**Affiliations:** 1 Department of Medical Oncology, National Cancer Center/National Clinical Research Center for Cancer/Cancer Hospital, Chinese Academy of Medical Sciences and Peking Union Medical College, Beijing 100021, China; 2 Department of Medical Oncology, First Hospital of Xi’an Jiaotong University, Xi’an 710061, China; 3 Department of Thoracic Surgery, Shanghai Chest Hospital, Shanghai Jiao Tong University, Shanghai 200030, China; 4 Department of Medical Oncology, First Affiliated Hospital of Xinxiang Medical University, Xinxiang 453100, China; 5 Department of Oncology, The First Affiliated Hospital of Zhengzhou University, Zhengzhou 450052, China; 6 Department of Oncology, The First Affiliated Hospital of Nanjing Medical University, Nanjing 210029, China; 7 Department of Medical Oncology, Jiangsu Cancer Hospital, Nanjing 210009, China; 8 Department of Medical Oncology, Shandong Cancer Hospital, Jinan 250117, China; 9 Department of Medical Oncology, Tianjin Medical University Cancer Institute and Hospital, National Clinical Research Center for Cancer, Key Laboratory of Cancer Prevention and Therapy, Tianjin, Tianjin’s Clinical Research Center for Cancer, Tianjin 300060, China; 10 Department of Biostatistics, School of Public Health, Nanjing Medical University, Nanjing 210029, China; 11 Department of Medical Oncology, Henan Cancer Hospital, Zhengzhou 450008, China; 12 Department of Oncology, Peking Union Medical College Hospital, Chinese Academy of Medical Sciences, Beijing 100021, China; 13 Department of Medical Oncology, Harbin Medical University Cancer Hospital, Harbin 150086, China; 14 Department of Gastroenterology, Affiliated Tumor Hospital, Xinjiang Medical University, Urumqi 830011, China; 15 Department of Thoracic Surgical Oncology, National Cancer Center/National Clinical Research Center for Cancer/Cancer Hospital, Chinese Academy of Medical Sciences and Peking Union Medical College, Beijing 100021, China

**Keywords:** Esophageal squamous cell carcinoma (ESCC), anlotinib, treatment-induced hypertension, prognostic predictor, antiangiogenesis

## Abstract

**Objective:**

In this post-hoc analysis, we evaluated anlotinib treatment-induced hypertension as a potential predictive factor of efficacy in esophageal squamous cell carcinoma (ESCC) patients.

**Methods:**

A total of 109 patients enrolled in the anlotinib group in a phase 2 trial were included. The tumor response was assessed by computed tomography at week 3, week 6, and then every 6 weeks until progressive disease was observed. The primary endpoint of the study was progression free survival (PFS). The secondary endpoints included overall survival (OS) and objective response rate (ORR).

**Results:**

In all patients, the median PFS was 3.02 months [95% confidence interval (CI): 2.63–3.65 months] and the OS was 6.11 months (95% CI: 4.40–7.79 months). The ORR was 7.34% (95% CI: 3.22%–13.95%). A total of 59 (54%) patients were diagnosed with treatment-induced hypertension (Group A), and the remaining patients (*n* = 50, 46%) were in Group B. Baseline prognostic factors were similar between the 2 groups. Patients in Group A had a longer PFS and OS and higher ORR. When stratifying patients using a previously known history of hypertension, treatment-induced hypertension was a predictor only for patients without previous hypertension, who had longer PFS [hazard ratio (HR): 0.40, 95% CI: 0.24–0.68] and OS (HR: 0.37, 95% CI: 0.21–0.67).

**Conclusions:**

We showed, for the first time, a correlation between treatment-induced hypertension and better prognoses in recurrent or metastatic ESCC patients treated with anlotinib, without a previously known history of hypertension. Treatment-induced hypertension may be a simple and low cost predictor for anlotinib antitumor efficacy in these patients, which may also reflect the intended target inhibition.

## Introduction

Esophageal cancer is the seventh most common malignancy, the sixth leading cause of cancer-related deaths worldwide, and the fourth leading cause of cancer-related mortality in China^1,2^. Esophageal squamous cell carcinoma (ESCC) is the most common subtype with a poor 5-year survival rate of only about 15%–25% in China^3^. Over past decades, recurrent or metastatic ESCC has been managed mainly using platinum plus paclitaxel or fluorouracil-based chemotherapy^4,5^. However, the long-term survival remains poor and needs to be improved.

Anlotinib is an oral small molecule tyrosine kinase inhibitor (TKI) targeting the vascular endothelial growth factor receptor (VEGFR) 1/2/3, fibroblast growth factor receptor 1–4, platelet-derived growth factor receptor (PDGFR) α/β, Ret, and c-Kit^6^. A phase III trial has shown that anlotinib improved the progression-free survival (PFS) and overall survival (OS) of patients with advanced non-small cell lung cancer (NSCLC)^7^. In ESCC, expression of VEGF is associated with angiogenesis, tumorigenesis, and progressive disease. Various co-expression patterns of VEGFR1/2/3 and PDGFR α/β at the transcriptional level were observed in ESCC^8^. Multitarget TKIs may therefore have anti-tumor efficacy in ESCC. In a previous multicenter, randomized, double-blind, placebo-controlled phase II trial (registration number: NCT02649361), anlotinib significantly improved the median PFS when compared with the placebo, in patients with recurrent or metastatic ESCC [3.02 *vs.* 1.41 months, hazard ratio (HR): 0.46, 95% confidence interval (CI): 0.32–0.66, *P* < 0.0001]^9^.

Treatment-induced hypertension has been proposed as a potential predictive factor of the clinical efficacy of antiangiogenic agents. Many retrospective analyses showed a correlation between treatment-related hypertension and better clinical outcomes in patients treated with anti-angiogenic agents with various malignancies, including NSCLC, gastrointestinal stromal tumor, renal cell carcinoma, colorectal carcinoma, and differentiated thyroid cancer^10–14^. In patients with different types of malignancies who received anlotinib, treatment-induced hypertension was also an independent predictive factor for better prognoses^15–18^. Currently, there has been no similar study in ESCC patients receiving anlotinib or any other antiangiogenic agents. In this post-hoc analysis, we showed that treatment-induced hypertension was a predictive factor for the efficacy of anlotinib treatment in recurrent or metastatic ESCC patients.

## Materials and methods

### Patients and treatments

This was a post-hoc analysis from a randomized, double-blind, placebo-controlled, phase II trial, which was conducted in 13 hospitals in China (Trial Registration No. NCT02649361). Eligible patients were 18–75 years of age, and had histologically confirmed recurrent or metastatic ESCC (stage IV) with at least one measurable lesion. A total of 109 patients enrolled in the anlotinib group were included in this post-hoc analysis. Patients received oral anlotinib (12 mg per day) in 3-week cycles (2 weeks on and 1 week off). Scheduled visits and computed tomography scans were performed on weeks 3, 6, and then every 6 weeks until disease progression was observed. The tumor responses were assessed based on the Response Evaluation Criteria In Solid Tumors (RECIST), version 1.1^19^. Safety data were documented during the treatment and first 30 days after the last administration of anlotinib. The investigators graded all adverse events according to the National Cancer Institute Common Terminology Criteria for Adverse Events (NCI-CTCAE, version 4.0). All participants were followed-up every 2 months for survival status after the last administration of anlotinib.

The ethics committee at each study hospital approved the study protocol and all amendments (Approval No. 15-125/1052). The trial was conducted in accordance with Good Clinical Practice guidelines and the tenets of the Declaration of Helsinki. Written informed consent was obtained from all patients before enrollment.

### Outcomes

The primary endpoint of the study was PFS, which was defined as the time from randomization to disease progression or death from any cause, whichever occurred first. The secondary endpoints were the OS (defined as the time from randomization to death from any cause) and ORR (the percentage of patients with a confirmed complete or partial response).

### Treatment-induced hypertension

Blood pressure (BP) was measured during each visit. According to NCI-CTCAE, version 4.0, treatment-induced hypertension was defined as systolic BP ≥ 120 mmHg and/or diastolic BP ≥ 80 mmHg without a known history of hypertension, or patients who required intensification of medication due to worsening of previously known hypertension, at any time after day 1 of treatment.

### Statistical analysis

The PFS and OS were estimated using the Kaplan-Meier method and compared between patients with (Group A) or without (Group B) treatment-induced hypertension using the log-rank test. Subgroup analysis was further conducted in patients with or without previous hypertension (history of hypertension disease). The ORR was compared between groups using Pearson’s chi-square or Fisher’s exact test, as appropriate. A two-tailed *P* < 0.05 was considered significant. SAS 9.2 software (SAS Institute, Cary, NC, USA) was used for statistical analyses.

## Results

Between January 6, 2016 and May 22, 2018, a total of 109 patients were recruited to receive anlotinib. By the data cutoff date of July 22, 2018, the median treatment duration was 2.56 months (range: 0.50–20.86 months). The investigator-assessed median PFS was 3.02 months (95% CI: 2.63–3.65 months) and the OS was 6.11 months (95% CI: 4.40–7.79 months). The ORR was 7.34% (95% CI 3.22%–13.95%).

A total of 59 (54%) patients were diagnosed with treatment-induced hypertension (Group A); the remaining patients (*n* = 50, 46%) were in Group B. There were no significant differences in most important baseline prognostic factors between Groups A and B (age, gender, ECOG performance status score, tumor differentiation, previous tumor surgery, previous chemotherapy, or previous hypertension; all, *P* > 0.05, **Table 1**). When stratifying patients according to previous hypertension, the baseline characteristics were also balanced (**Supplementary Table S1**; all, *P* > 0.05). The BP of all patients with treatment-induced hypertension was controllable by prescribing or adjusting antihypertensive medications.

**Table 1:** Baseline characteristics

Characteristics	Group A (*n* = 59)	Group B (*n* = 50)
Age (years)		
≥ 65	20 (33.9%)	15 (30.0%)
< 65	39 (66.1%)	35 (70.0%)
Gender		
Male	43 (72.9%)	43 (86.0%)
Female	16 (27.1%)	7 (14.0%)
ECOG performance status score		
0	8 (13.6%)	6 (12.0%)
1	49 (83.1%)	38 (76.0%)
2	2 (3.4%)	6 (12.0%)
Tumor differentiation		
Undifferentiated or poorly differentiated	16 (27.1%)	18 (36.0%)
Moderately or well differentiated	43 (72.9%)	32 (64.0%)
Previous tumor surgery		
Yes	38 (64.4%)	34 (68.0%)
No	21 (35.6%)	16 (32.0%)
Previous chemotherapy		
One line	19 (32.2%)	20 (40.0%)
Two or more lines	40 (67.8%)	30 (60.0%)
Previous hypertension		
Yes	16 (27.1%)	17 (34.0%)
No	43 (72.9%)	33 (66.0%)

**Figure 1:**
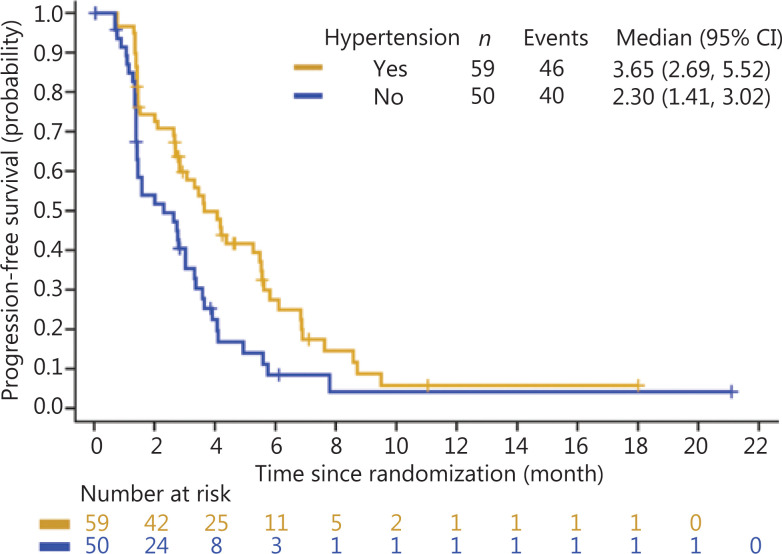
Kaplan-Meier curve of progression-free survival of treatment-induced hypertension in the anlotinib group.

**Figure 2:**
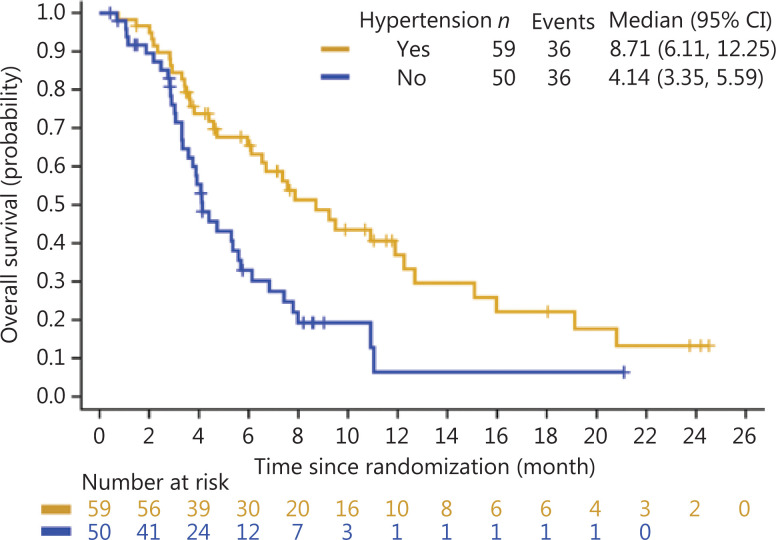
Kaplan-Meier curve of the overall survival of treatment- induced hypertension in the anlotinib group.

Overall, treatment-induced hypertension was associated with longer PFS and OS. The median PFS was 3.65 months in Group A (95% CI: 2.69–5.52 months) *vs.* 2.30 months in Group B (95% CI: 1.41–3.02 months) (HR: 0.56, 95% CI: 0.37–0.86) (**Figure 1**). The median OS was 8.71 months in Group A (95% CI: 6.11–12.25 months) and 4.14 months in Group B (95% CI: 3.35–5.59 months) (HR: 0.47, 95% CI: 0.29–0.76) (**Figure 2**). Treatment-induced hypertension was also associated with improved clinical outcomes. The ORR in Groups A and B were 10.20% and 4.00%, respectively (*P* = 0.048).

However, when stratifying patients according to a history of hypertension disease, similar findings were only observed in patients without previous hypertension (**Table 2**). In this subgroup of patients, the median PFS was 4.17 months in Group A (95% CI: 2.63–5.55 months) *vs.* 1.41 months in Group B (95% CI: 1.35–2.76 months) (HR: 0.40, 95% CI: 0.24–0.68), the median OS was 8.71 months in Group A (95% CI: 5.95–15.08 months) and 4.07 months in Group B (95% CI: 3.32–5.59 months) (HR: 0.37, 95% CI: 0.21–0.67), the ORR in Groups A and B were 9.30% (95% CI: 2.59%–22.14%) and 3.03% (95% CI: 0.08%–15.76%), respectively, with *P* = 0.3806.

**Table 2:** Progression-free survival, overall survival, and the objective response rate according to previous hypertension

Previous hypertension	Treatment-induced hypertension	*n*	Median (95% CI)	HR^a^/OR^b^ (95% CI)
OS^c^				
Yes	Group A	16	9.23 months (3.61, 12.25)	0.74 (0.31, 1.77)
	Group B	17	5.36 months (3.02, 11.04)	
No	Group A	43	8.71 months (5.95, 15.08)	0.37 (0.21, 0.67)
	Group B	33	4.07 months (3.32, 5.59)	
PFS^d^				
Yes	Group A	16	3.61 months (2.69, 6.83)	0.86 (0.39, 1.92)
	Group B	17	3.02 months (2.00, 5.59)	
No	Group A	43	4.17 months (2.63, 5.55)	0.40 (0.24, 0.68)
	Group B	33	1.41 months (1.35, 2.76)	
ORR^e^				
Yes	Group A	16	12.5% (1.55%, 38.35%)	0.44 (0.04, 5.36)
	Group B	17	5.88% (0.15%, 28.69%)	
No	Group A	43	9.30% (2.59%, 22.14%)	0.30 (0.03, 2.86)
	Group B	33	3.03% (0.08%, 15.76%)	

^a^HR, hazard ratio; ^b^OR, odds ratio; ^c^OS, overall survival; ^d^PFS, progression-free survival; ^e^ORR, objective response rate.

In Group A, anlotinib-induced hypertension involved grades 1–3 according to NIC-CTCAE, version 4.0, in which the case numbers of each grade were 13, 29, and 17, respectively. There was no grade 4 or 5 hypertension. The percentage of dose reduction and discontinuation of treatment related to hypertension were both 3.39% (2/59).

## Discussion

In this study, treatment-induced hypertension was correlated with longer PFS/OS and better ORR in recurrent or metastatic ESCC patients treated with anlotinib, who did not have a history of hypertension disease. Our results suggested that treatment-induced hypertension could be a potential predictor for anlotinib efficacy in these patients.

According to earlier findings, the development or worsening of hypertension was a predictive factor for responses to anlotinib and favorable outcomes in NSCLC^15,16^, soft tissue sarcoma^17^, and medullary thyroid carcinoma^18^ patients. In the present study, we found that anlotinib treatment-induced hypertension was also associated with significantly improved clinical outcomes in recurrent or metastatic ESCC. Patients with treatment-induced hypertension had a 1.35 and 4.57 months longer median PFS and OS, respectively, and 6.2% higher ORR. The improvements in PFS and OS in the overall population were significant with HRs of 0.56 and 0.47, respectively. However, when stratifying patients with/without previous hypertension, improvements were only observed in patients without previous hypertension (HR for PFS and OS: 0.40 and 0.37, respectively). It was unclear why treatment-induced hypertension failed to predict outcomes in patients with a previous history of hypertension; however, the relatively small case number (33 in total; 16 and 17 in Groups A and B, respectively) may not have been sufficient to demonstrate any meaningful difference. Nevertheless, these results suggested that treatment-induced hypertension could be a potential factor for predicting better prognosis in recurrent or metastatic ESCC patients without a known history of hypertension.

The underlying mechanism of the correlation between treatment-induced hypertension and better prognoses in patients treated with antiangiogenic therapy requires further study. Antagonism of the VEGF pathway has been shown to promote hypertension by decreasing nitric oxide production, which leads to vasculature constriction and reduction in sodium ion renal excretion. A recent study reported a correlation involving a significant reduction of capillary density, changes in vascular morphology, and significantly prolonged PFS in renal cell carcinoma patients treated with sunitinib^20^. The authors hypothesized that the more robust antiangiogenic effect may have been linked to the susceptibility of both normal blood vessels and tumor vessels, and may have led to both development of hypertension and enhanced clinical outcomes^20^. Other studies suggested that the susceptibility of antiangiogenic agent treatment-induced hypertension may be associated with certain single nucleotide polymorphisms (SNPs) in the genes of the VEGF pathway^21–25^. Some genotypes of these SNPs were also associated with prognosis in advanced breast cancer^24^ or colorectal cancer patients^25^ treated by bevacizumab, and in clear cell renal cell carcinoma patients treated by sunitinib^23^.

Treatment-induced hypertension with anlotinib ranged from 39% to 68% in other carcinomas, such as NSCLC^16^, soft tissue sarcoma^17^, medullary thyroid carcinoma^18^, small-cell lung carcinomaa^26^, and renal cell carcinoma^27,28^. Our results are consistent with previous findings (54%). In the present study, anlotinib-induced hypertension may be successfully controlled in most cases without a reduction in anlotinib dosing or interruptions in treatment. Similar findings have been reported with other inhibitors of the VEGF signaling pathway in other tumor types, such as the treatment of sunitinib in advanced renal cell carcinoma^29^.

Our study was limited by its retrospective nature, so the findings need to be validated in further prospective studies. Moreover, this study included Chinese patients only. Whether these results could be reproduced in other patient populations is still unclear.

## Conclusions

We had shown, for the first time, a correlation between treatment-induced hypertension and better prognoses in recurrent or metastatic ESCC patients treated with anlotinib, who did not have a previously known history of hypertension. Treatment-induced hypertension may be a simple and low cost predictor for anlotinib antitumor efficacy in these patients, which may also reflect the intended target inhibition.
