# Supplementary material for: The relationship between treatment-induced hypertension and efficacy of anlotinib in recurrent or metastatic esophageal squamous cell carcinoma
Source: Cancer Biol Med. 2021 Jun 15;18(2):562–8. doi: 10.20892/j.issn.2095-3941.2020.0187 (PMC8185854; doi:10.20892/j.issn.2095-3941.2020.0187)
Supplement: Supplementary file 1 [file cbm-18-562-s001.pdf]

Supplementary material

Table 1 Baseline characteristics according to previous hypertension

Previous hypertension	Yes		No	
	Group A (*n* = 16)	Group B (*n* = 17)	Group A (*n* = 43)	Group B (*n* = 33)
Age				
≥ 65	5 (31.25%)	8 (47.06%)	15 (34.88%)	7 (21.21%)
65	11 (68.75%)	9 (52.94%)	28 (65.12%)	26 (78.79%)
Gender				
Male	13 (81.25%)	16 (94.12%)	30 (69.77%)	27 (81.82%)
Female	3 (18.75%)	1 (5.88%)	13 (30.23%)	6 (18.18%)
ECOG				
0	2 (12.50%)	1 (5.88%)	6 (13.95%)	5 (15.15%)
1	14 (87.50%)	14 (82.35%)	35 (81.40%)	24 (72.73%)
2	0 (0)	2 (11.76%)	2 (4.65%)	4 (12.12%)
Tumor differentiation				
Undifferentiated or poorly differentiated	3 (18.75%)	7 (41.18%)	13 (30.23%)	11 (33.33%)
Moderately or well differentiated	13 (81.25%)	10 (58.82%)	30 (69.77%)	22 (66.67%)
Previous tumor surgery				
Yes	14 (87.50%)	11 (64.71%)	24 (55.81%)	23 (69.70%)
No	2 (12.50%)	6 (35.29%)	19 (44.19%)	10 (30.30%)
Previous chemotherapy				
One line	7 (43.75%)	4 (23.53%)	12 (27.91%)	16 (48.48%)
Two or more lines	9 (56.25%)	13 (76.47%)	31 (72.09%)	17 (51.52%)
